# Exploring the Cardiovascular Safety Profile of Ibuprofen: Insights from EudraVigilance Database

**DOI:** 10.3390/ph18071045

**Published:** 2025-07-17

**Authors:** Cristina Anamaria Buciuman, Carmen Maximiliana Dobrea, Anca Butuca, Adina Frum, Felicia Gabriela Gligor, Octavia Gligor, Laura Grațiela Vicaș, Claudiu Morgovan

**Affiliations:** 1 Faculty of Pharmacy, Carol Davila University of Medicine and Pharmacy, 6 Traian Vuia Str., 020956 Bucharest, Romania; buciuman.apetrii@drd.umfcd.ro (C.A.B.); lvicas@uoradea.ro (L.G.V.); 2 Preclinical Department, Faculty of Medicine, “Lucian Blaga” University of Sibiu, 2A Lucian Blaga Str., 550169 Sibiu, Romania; carmen.dobrea@ulbsibiu.ro (C.M.D.); anca.butuca@ulbsibiu.ro (A.B.); adina.frum@ulbsibiu.ro (A.F.); felicia.gligor@ulbsibiu.ro (F.G.G.); claudiu.morgovan@ulbsibiu.ro (C.M.); 3 Faculty of Medicine and Pharmacy, University of Oradea, 10, 1 December Square, 410073 Oradea, Romania

**Keywords:** ibuprofen safety, pharmacovigilance, EudraVigilance, cardiovascular toxicity, disproportionality analysis

## Abstract

**Background**: Ibuprofen is one of the most accessible non-steroidal anti-inflammatory drugs (NSADs), exhibiting non-selective reversible inhibition on COX-1 and COX-2. A series of common adverse reactions have been mentioned through the years: gastrointestinal (gastritis, ulceration, hemorrhage, or perforation), renal, hematologic, and cardiovascular. **Objective**: The aim of this study was to assess the real-world impact of ibuprofen regarding cardiovascular safety, utilizing an established pharmacovigilance database. **Methods**: Descriptive and disproportionality-based methods were used. Forty specific descriptors of cardiovascular effects were selected. Eight other NSADs and the combination of ibuprofen and pseudoephedrine were used as comparators. **Results**: A total of 58,760 cases were identified as being associated with ibuprofen in EudraVigilance. Stroke was reported for ibuprofen with a lower probability compared with etoricoxib (ROR: 0.34; 95% CI: 0.21–0.55), celecoxib (ROR: 0.07; 95% CI: 0.06–0.10), meloxicam (ROR: 0.25; 95% CI: 0.14–0.43), acetylsalicylic acid (ROR: 0.07; 95% CI: 0.05–0.09), and ibuprofen/pseudoephedrine (ROR: 0.11; 95% CI: 0.05–0.25). Thrombosis was reported for ibuprofen with a higher probability only relative to ketoprofen (ROR: 2.95; 95% CI: 1.71–5.09). Hypertension was reported for ibuprofen as being more probable than for acetylsalicylic acid (ROR: 1.58; 95% CI: 1.43–1.76). Myocardial infarction was reported as being more probable for ibuprofen than ketoprofen (ROR: 2.31; 95% CI: 1.57–3.40) or nimesulide (ROR: 2.43; 95% CI: 1.25–4.73). **Conclusions**: Overall, according to our study, the probability of reported cardiovascular adverse reactions is lower than those determined for the rest of the NSAIDs; however, taking into consideration the inherent limitations of the study, further clinical investigations would contribute to a better understanding of the cardiovascular safety of ibuprofen.

## 1. Introduction

Ibuprofen [2-(4-isobutylphenyl) propionic acid)-C_3_H_18_O_2_] is associated with the non-steroidal anti-inflammatory (NSAID) group. It is considered the first of the propionic class and was derived from propionic acid. Ibuprofen was developed in 1960 and patented in 1961. It was authorized on the market, as a primary indication, for rheumatoid arthritis in 1969 in the United Kingdom and in 1974 in the United States. It is administered as a racemic mixture. After administration, the R-enantiomer undergoes interconversion to the S-enantiomer in vivo under the activity of the alpha-methylacyl-CoA racemase. It is known that the S-enantiomer is capable of inducing a stronger pharmacological activity than the R-enantiomer [1,2].

**Figure 1:**
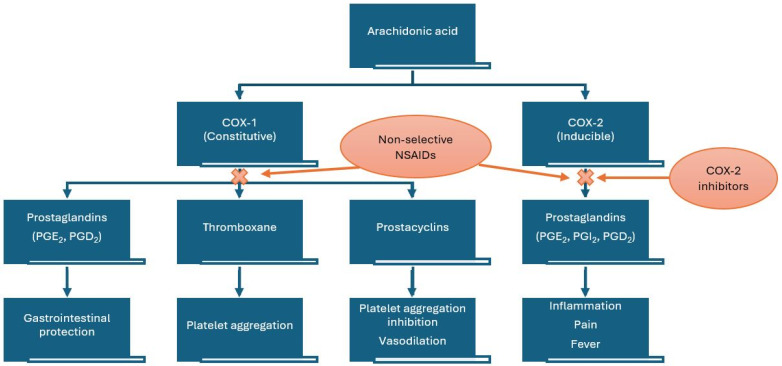
Mechanism of action of non-steroidal anti-inflammatory drugs (NSAIDs). COX—cyclooxygenase.

Ibuprofen has as its main mechanism of action the non-selective reversible inhibition of the cyclooxygenase enzymes COX-1 and COX-2. The cyclooxygenase pathway results in the conversion of arachidonic acid to prostaglandins (PGs), prostacyclin (PGI_2_), and thromboxanes (Tx) (Figure 1). It consists of three distinct isoforms: COX-1 (PGH synthase), COX-2, and COX-3.

Because of its mechanism of action ibuprofen alienates, through competition for the NSAID binding site of COX-1 in platelets, the cardioprotective effect of low-dose acetylsalicylic acid (AAS). Due to its reversible inhibition, in platelets, of COX-1, ibuprofen has a transient antiplatelet effect for 1 h during the 8 h dosing interval, which may result in increased bleeding risk when it is administered together with other anticoagulant or antiplatelet agents [3].

In the specific case of ibuprofen, COX-1 is inhibited approximately 2.5 times more strongly than COX-2 [4].

Ibuprofen has a short half-life (t_½_ = 2–4 h). Thus, in order to provide an adequate analgesic effect, it has to be administered frequently (3–4 times per day). Due to the fact that it is not a very potent COX inhibitor, high doses are required, and wide fluctuations of plasma concentrations are noted even under steady-state conditions [5].

Among the common adverse reactions associated with ibuprofen, we enumerate gastrointestinal, renal, hematologic, and cardiovascular adverse effects (e.g., hypertension, heart failure, myocardial infarction, and stroke) [4]. The cardiovascular risk from nonselective NSAIDs is considered to be caused by inhibition of PGI_2_, which increases platelet reactivity, together with inhibition of platelet COX-1. In order to avoid the side effects linked to the generic inhibition of COX enzymes, alongside the non-selective NSAIDS, selective COX-2 inhibitors have been authorized. Their main advantage refers to the lack of gastrointestinal side effects, but a greater risk of thrombotic cardiovascular events has been linked to higher ratios of COX-2 to COX-1 inhibition, and this is particularly important when comparing NSAIDs with selective COX-2 inhibitors, coxibs. These side effects manifest especially in association with high doses and long-term treatments [6,7,8]. NSAIDs also have effects on blood pressure (BP) and demonstrate interference with antihypertensive drugs, particularly angiotensin-converting enzyme inhibitors and angiotensin receptor blockers [9].

Higher COX-2 receptor occupancy is associated with an increased cardiovascular risk from NSAIDs, while higher COX-1 receptor occupancy is linked to lower cardiovascular risk [10]. The FDA (Food and Drug Administration) issued a warning in 2005 stating that NSAIDs like ibuprofen and naproxen may increase the risk of having a heart attack or stroke. Moreover, in July 2015, the FDA further strengthened this warning [11]. Small increases in the risks of heart attacks and strokes in patients taking high doses of ibuprofen (>2400 mg/day) have been established by EMA, as well [12].

Adverse drug reactions (ADRs) have to be reported by stakeholders (healthcare professionals, patients, and manufacturers) and monitored by medical regulatory agencies. In the European Union, the European Medicines Agency (EMA) coordinates the EU pharmacovigilance system and operates services and processes to support pharmacovigilance. EudraVigilance (EV) is the system utilized for managing and analyzing information on suspected adverse reactions to medicines that have been authorized or are being studied in clinical trials in the European Economic Area (EEA) [13]. Vigibase (the WHO global database) and FAERS (the US Food and Drug Administration adverse reporting system) are also used to manage suspected adverse reactions.

Due to the widespread use of ibuprofen, the aim of this study was to explore the ADRs related to its administration by analyzing reports from the EudraVigilance da-tabase, focusing on cardiovascular side effects. A disproportionality analysis was also carried out to better understand the patterns of the main cardiovascular events reported for ibuprofen [14,15,16,17,18,19,20,21,22,23,24].

## 2. Results

### 2.1. Descriptive Analysis

**Figure 2:**
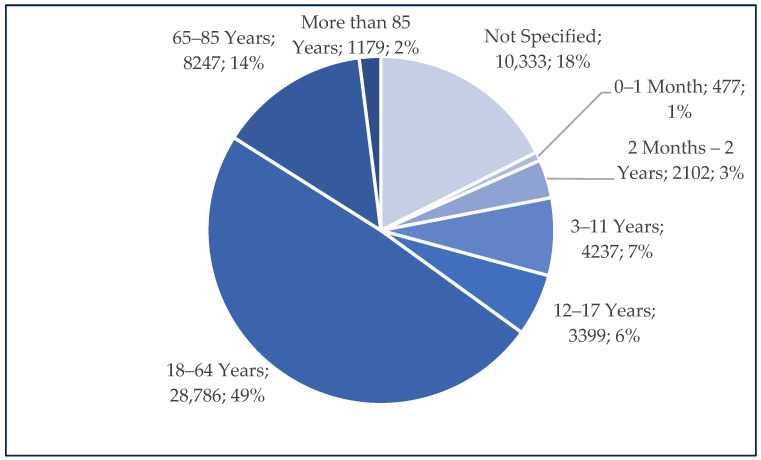
Distribution of reported age groups (n = 58,760).

Among the total number of cases reported for ibuprofen use (n = 58,760), 49% (n = 28,796) were related to patients aged 18–64 years. Similar frequencies of Individual Case Safety Reports (ICSRs) were registered in groups comprising patients under 18 years of age (n = 10,215; 17%) and, respectively, those over 65 years of age (n = 9426; 16%) (Figure 2).

A higher frequency of cases was registered for women (n = 32,343; 55%), compared to men (n = 22,911; 39%). Small differences regarding the origins of reports were observed. Thus, 53% of cases originated in European Economic Area (EEA) countries (n = 31,073) and 47% (n = 27,687) in non-EEA countries. Most cases were reported by healthcare professionals (HPs) (n = 43,654; 74%) and only 25% of cases were reported by respondents who were not healthcare professionals (n = 14,591).

**Figure 3:**
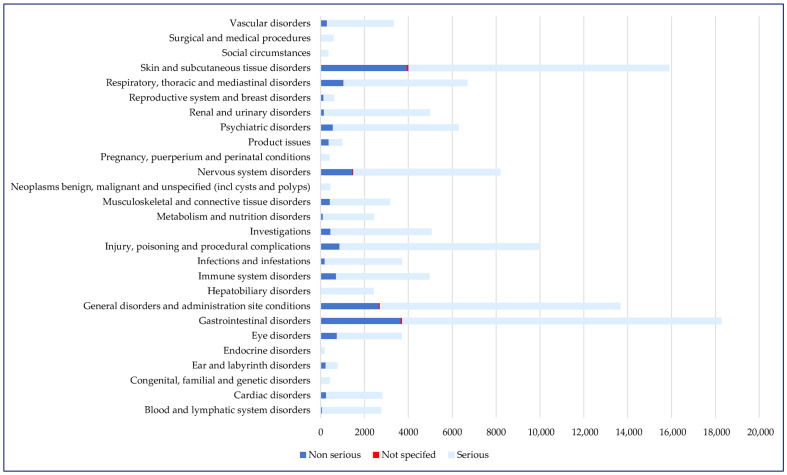
Distribution of cases by SOC and seriousness (n = 122,726). SOC—System Organ Class.

Regarding the distribution of cases according to System Organ Classes (SOCs), the most frequently reported were gastrointestinal disorders (n = 18,265) and skin and subcutaneous tissue disorders (n = 15,885), but the frequency values of serious cases in these SOCs were among the lowest (74.7% for skin disorders and 79.6% for gastrointestinal disorders). Although the numbers of reports associated with cardiac disorders (n = 2801) and vascular disorders (n = 3327) were smaller, about 90% of these were declared to be serious (Figure 3). Other SOCs have a broader scope, as defined by Medical Dictionary for Regulatory Activities (MedDRA). “Investigations” includes reports referring to abnormal tests, such as increased alanine aminotransferase levels, leucopenia, and electrocardiogram abnormalities. “Social circumstances” groups social determinants impacting treatment or disease management, such as inadequate access to care or support systems, hospital admission driven by social factors rather than medical necessity, or pregnancy occurring in contexts of significant social vulnerability (e.g., poverty, domestic violence, homelessness, or lack of prenatal care) [17].

**Figure 4:**
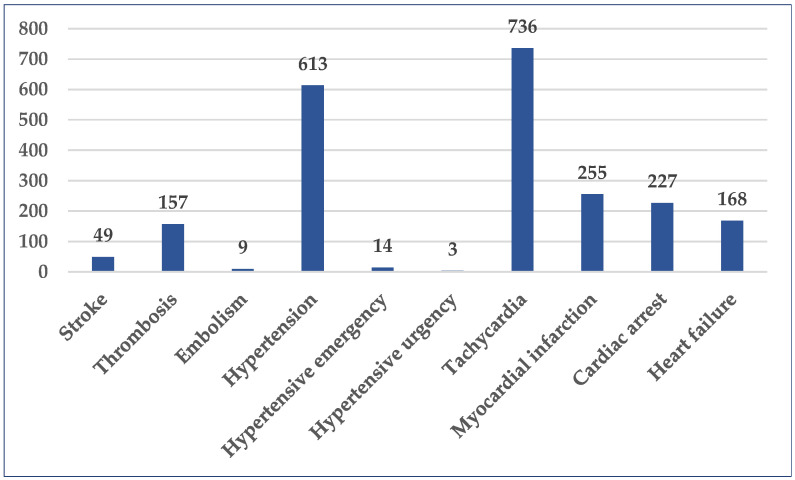
Distribution of cases by selected SMQ (n = 2231). SMQ—Standardized MedDRA Queries.

A total of 10 Standardized MedDRA Queries (SMQ) associated with cardiovascular disorders were selected. High numbers of cases were related to tachycardia (n = 736, 1.25%), hypertension (n = 613, 1.04%), myocardial infarction (n = 255, 0.43%), cardiac arrest (n = 227, 0.39%), heart failure (n = 168, 0.29%), and thrombosis (n = 157, 0.27%). The blood pressure was severely elevated (≥180/120 mmHg) in seventeen (0.03%) cases, out of which only three didn’t present clinical signs of acute target organ damage (hypertensive urgency). The number of cases reported for stroke, another medical emergency, was 49 (0.08%) (Figure 4).

### 2.2. Disproportionality Analysis

**Figure 5:**
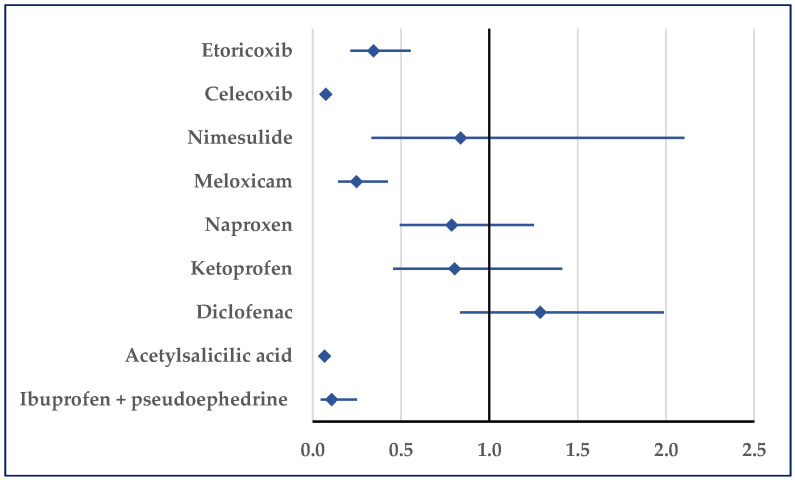
Reporting odds ratio for cases related to stroke. An ROR greater than 1 denotes a higher probability of reporting.

Stroke was reported for ibuprofen with a lower probability compared with etoricoxib (reporting odds ratio (ROR): 0.34; 95% confidence interval (CI): 0.21–0.55), celecoxib (ROR: 0.07; 95% CI: 0.06–0.10), meloxicam (ROR: 0.25; 95% CI: 0.14–0.43), acetylsalicylic acid (ROR: 0.07; 95% CI: 0.05–0.09), and ibuprofen/pseudoephedrine (ROR: 0.11; 95% CI: 0.05–0.25) (Figure 5, Table S1). No statistically significant differences were observed in comparisons to the other selected NSAIDs.

**Figure 6:**
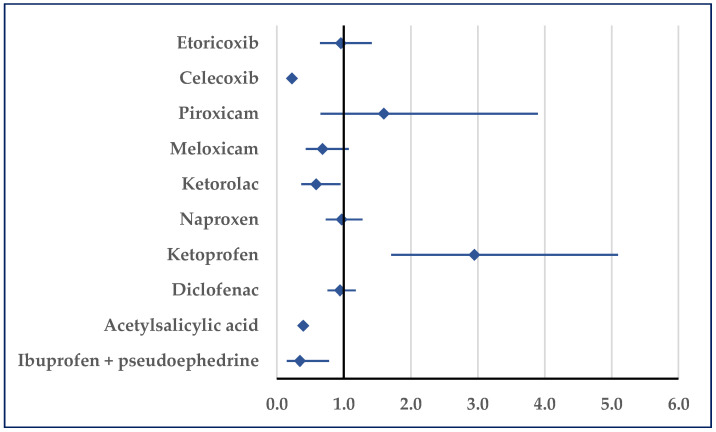
Reporting odds ratio of cases related to thrombosis. An ROR greater than 1 denotes a higher probability of reporting.

Thrombosis was reported for ibuprofen with a higher probability only relative to ketoprofen (ROR: 2.95; 95% CI: 1.71–5.09). On the contrary, for ibuprofen a lower probability of reported thrombosis was noticed in comparison to acetylsalicylic acid (ROR: 0.39; 95% CI: 0.33–0.47), ketorolac (ROR: 0.59; 95% CI: 0.37–0.95), celecoxib (ROR: 0.22; 95% CI: 0.19–0.27), and ibuprofen/pseudoephedrine (ROR: 0.34; 95% CI: 0.15–0.78) (Figure 6).

**Figure 7:**
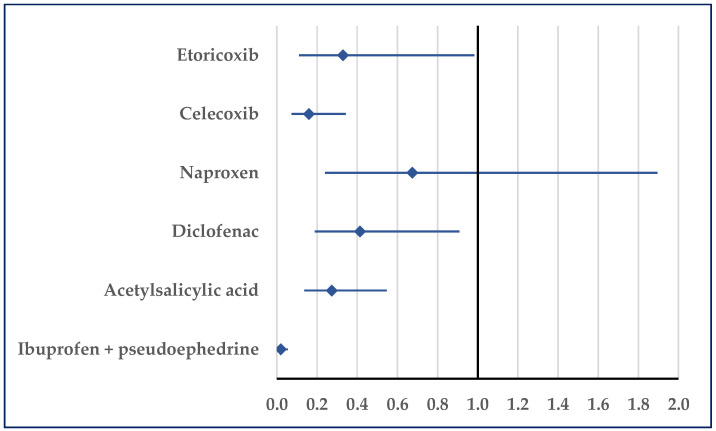
Reporting odds ratio of cases related to embolism. An ROR greater than 1 denotes a higher probability of reporting.

Figure 7 presents the probability of reporting embolism. Thus, a disproportionate number of reports was noticed in comparison to etoricoxib (ROR: 0.33; 95% CI: 0.11–0.98), celecoxib (ROR: 0.16; 95% CI: 0.07–0.34), diclofenac (ROR: 0.41; 95% CI: 0.19–0.91), acetylsalicylic acid (ROR: 0.27; 95% CI: 0.14–0.55), and ibuprofen/pseudoephedrine (ROR: 0.02; 95% CI: 0.01–0.06) (Figure 7).

**Figure 8:**
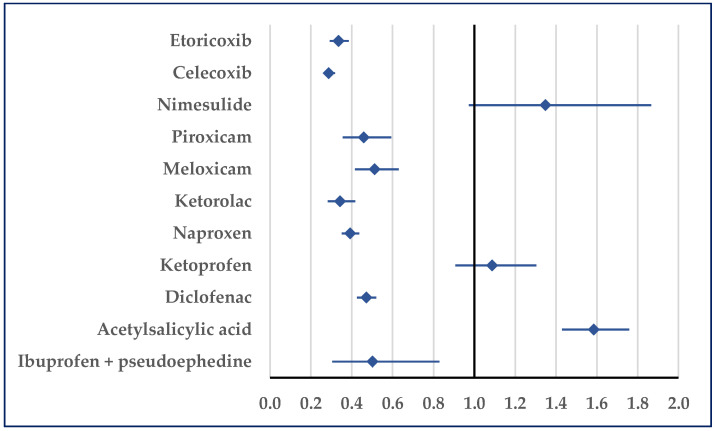
Reporting odds ratio of cases related to hypertension. An ROR greater than 1 denotes a higher probability of reporting.

Hypertension was reported for ibuprofen as being more probable than for acetylsalicylic acid (ROR: 1.58; 95% CI: 1.43–1.76). Compared with ketoprofen and nimesulide, a disproportionate number of cases was not registered, but by comparison with all other NSAIDs a lower probability of reporting was noticed for ibuprofen (Figure 8). Regarding hypertensive crisis, a disproportionate result was observed only in comparison with acetylsalicylic acid in hypertensive emergencies. Thus, for ibuprofen, a higher probability of reported hypertensive emergencies was noticed (ROR: 5.2472; 95% CI: 2.02–13.66).

**Figure 9:**
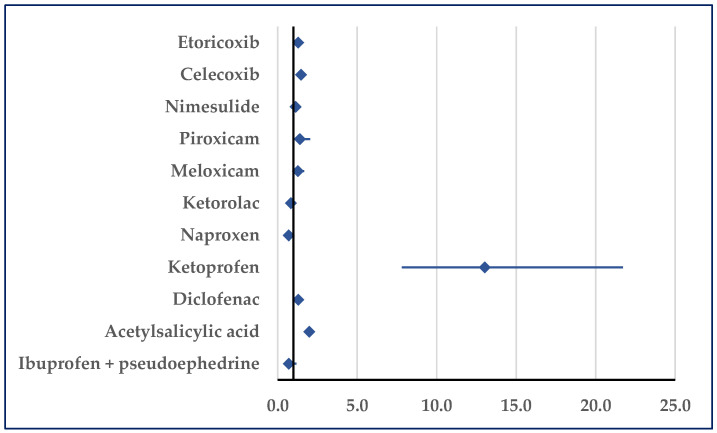
Reporting odds ratio of cases related to tachycardia. An ROR greater than 1 denotes a higher probability of reporting.

Reports of tachycardia associated with ibuprofen registered a higher probability than the values associated with acetylsalicylic acid (ROR: 1.98; 95% CI: 1.79–2.19), diclofenac (ROR: 1.31; 95% CI: 1.17–1.47), ketoprofen (ROR: 13.02; 95% CI: 7.81–21.72), celecoxib (ROR: 1.47; 95% CI: 1.26–1.72), and etoricoxib (ROR: 1.29; 95% CI: 1.05–1.58). Only in comparison with naproxen was a lower probability of reported tachycardia registered for ibuprofen (ROR: 0.70; 95% CI: 0.62–0.78) (Figure 9).

**Figure 10:**
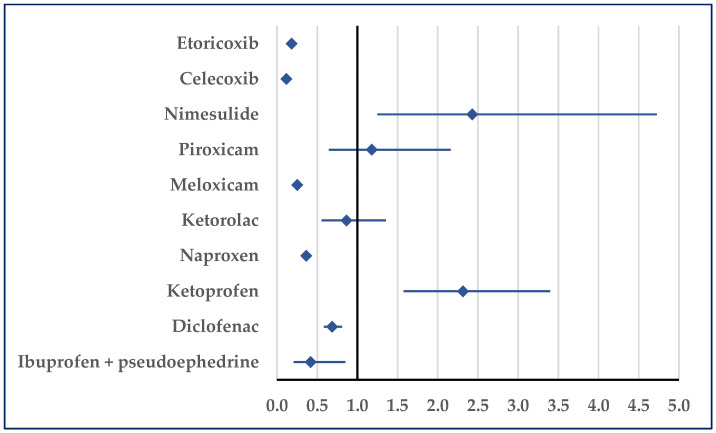
Reporting odds ratio of cases related to myocardial infarction. An ROR greater than 1 denotes a higher probability of reporting.

Myocardial infarction was reported as being more probable for ibuprofen than for ketoprofen (ROR: 2.31; 95% CI: 1.57–3.40) or nimesulide (ROR: 2.43; 95% CI: 1.25–4.73). No disproportionate result was noticed in comparisons with ketorolac and piroxicam, but a lower probability of reporting was noticed in comparisons with the other NSAIDs (Figure 10).

**Figure 11:**
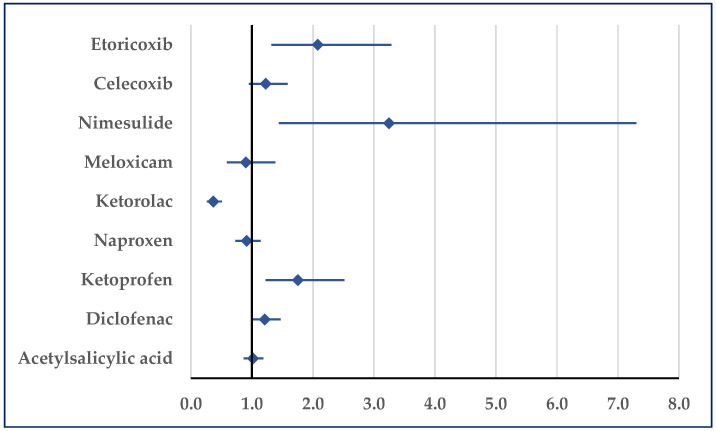
Reporting odds ratio of cases related to cardiac arrest. An ROR greater than 1 denotes a higher probability of reporting.

When compared to ketorolac (ROR: 0.37; 95% CI: 0.26–0.51), ibuprofen was associated with a reduced probability of cardiac arrest reports, but it was associated with a higher probability compared with ketoprofen (ROR: 1.75; 95% CI: 1.22–2.52), nimesulide (ROR: 3.24; 95% CI: 1.44–7.30), and etoricoxib (ROR: 2.08; 95% CI: 1.32–3.29) (Figure 11).

**Figure 12:**
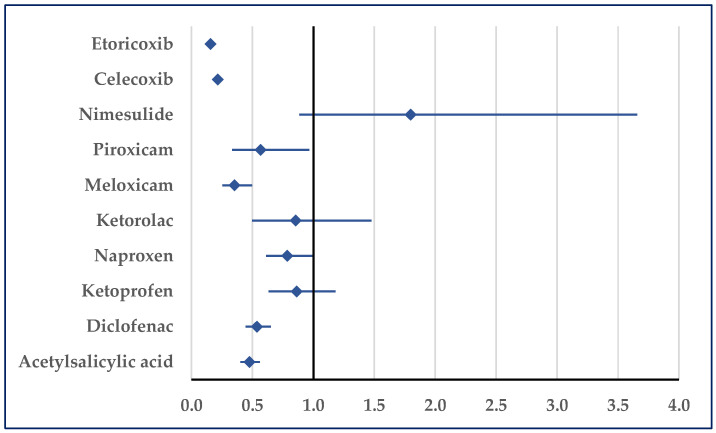
Reporting odds ratio of cases related to heart failure. An ROR greater than 1 denotes a higher probability of reporting.

According to Figure 12, heart failure was reported for ibuprofen with a lower probability than those for etoricoxib (ROR: 0.16; 95% CI: 0.13–0.19), celecoxib (ROR: 0.22; 95% CI: 0.18–0.26), piroxicam (ROR: 0.57; 95% CI: 0.33–0.96), meloxicam (ROR: 0.35; 95% CI: 0.25–0.50), diclofenac (ROR: 0.54; 95% CI: 0.44–0.65), and acetylsalicylic acid (ROR: 0.47; 95% CI: 0.40–0.56) (Figure 12).

## 3. Discussion

Our study assessed the cardiac toxicity of ibuprofen, based on real-world evidence, by focusing on certain medical conditions that are life threatening (i.e., stroke, cardiac arrest, myocardial infarct, embolism, etc.). All of these SMQs were identified in the reports uploaded to the EV database and were assessed in this retrospective study conducted on data ranging across a period of over 20 years. Even though these risks were identified, the overall probability of reported cardiac toxicity is lower for ibuprofen in comparison to the other NSAIDs used as comparators in the present study.

### 3.1. Descriptive Analysis

Our study revealed that approximately 50% of the ICSRs concerning ibuprofen were reported in the 18–64 age group, with the remaining 50% being distributed between the group comprising those under 18 and the group comprising those over 65. As Martinelli M. et al. reported in a national study conducted in Italy, pediatricians are generally aware of ibuprofen-prescribing patterns, and most of the reported ADRs in children were mild [25]. Moreover, ibuprofen dosing in the pediatric population is regulated differently across different countries. Thus, in North American and South American countries, ibuprofen can be administered to children beginning at the age of 6 months. On the other hand, in Europe, ibuprofen can be administered orally from the age of 3 months [26,27]. Furthermore, the study CPI-CL-022 investigated the pharmacokinetic elements and safety of intravenous ibuprofen in neonates aged birth to 6 months, setting the proper IV dose as 10 mg/kg, with a maximum of a 100 mg single dose [28]. In contrast, in elderly patients—who frequently present multiple comorbidities—ibuprofen dosing and its potential interactions require careful monitoring, with attention given to the balancing of risks and benefits [29]. Safety concerns regarding the administration of NSAIDs were addressed in a large multicenter phase IV study (PRECISION) performed between October 2006 and April 2016. The main outcome was the determination of a major risk of toxicity associated with NSAIDs (i.e., ibuprofen, celecoxib, naproxen). Among 24,081 patients with osteoarthritis or rheumatoid arthritis at moderate or high cardiovascular risk, 8040 patients were assigned to ibuprofen. Among the patients treated with ibuprofen, a group with a mean age of 63.2 years, 5.3% experienced major toxicity during the follow-up period [30].

The present study showed that a 55% of cases were reported for women, versus 39% for men. The proportion of females included in the ibuprofen arm of the PRECISION study was 64.4% [30]. Whether there are sex-based differences in the relevant pharmacokinetics, and thus with respect to these adverse reactions or their rate of reporting, remains to be discussed. Ochoa D. et al. concluded, in a study including 122 healthy volunteers and evaluating the effects of polymorphisms in CYP2C9 and CYP2C8 and sex on the pharmacokinetics of the enantiomeric forms of ibuprofen, that sex was associated with differences in the pharmacokinetics of both enantiomeric forms, with women showing a higher clearance rate [31]. Another study, conducted by Barden et al., concluded that there is no clinically meaningful difference in the efficacy rates of ibuprofen 400 mg associated with men and women experiencing moderate to severe postoperative pain [32]. Sex-based differences in pain therapy include many biological and social aspects that need further tackling. The disparities between the results of these studies (some concluding that ibuprofen resulted in significantly better pain reduction in men than in women, others that the pain-lowering effect of ibuprofen was comparable in men and women) can be explained by differences in nociceptive mechanisms in the experimental pain models and various factors influencing pain perception in clinical practice. The potential sex difference relating to nociception might be connected to estrogenic effects on the activity of the nervous system, resulting in improved transmission of pain impulses [33].

Concerning the geographic implications, 53% of ICSRs included in this study were reported in countries within the EEA (European Economic Area). EEA countries have developed strong tools to encourage adverse-reaction reporting, and a well-established database has been created to support continuous efforts to improve data management regarding the safe use of medicine [34]. Also, 47% of reports were initiated in non-EEA countries, which have individual country-based systems of collecting RA.

Although ADRs reported are mainly from the gastrointestinal (GI) and dermatological ranges, the ratios of serious reports (<80%) associated with these reactions are not as high as those reported for cardiac and vascular serious adverse reactions (>90%) [27,30,35,36,37,38,39,40,41,42].

### 3.2. Disproportionality Analysis

One famous study covering the cardiovascular safety of celecoxib, naproxen, and ibuprofen with respect to their use for arthritis is the PRECISION study. At moderate doses, celecoxib was found to be non-inferior to ibuprofen or naproxen, relative to cardiovascular safety [37]. Although this finding is informative, the cardiovascular safety findings derived from the PRECISION study cannot be extrapolated to the safety of the over-the-counter pain relievers ibuprofen and naproxen, given that the doses used were higher (ibuprofen to 800 mg three times a day and naproxen to 500 mg twice a day). PRECISION-ABPM, a sub-study of the PRECISION study, shows that the nonselective NSAID ibuprofen is associated with a significant rise in systolic BP and a higher rate of hypertension development, compared to the selective COX-2 inhibitor celecoxib [9,37]. According with these results, our study reveals that a higher probability of reported hypertension emergencies can be observed for ibuprofen, in comparison to acetylsalicylic acid. A case crossover study included residents of Denmark ≥ 18 years (n = 59,150), including population-based subsets associated with gout and having experienced a cardiovascular event (myocardial infarction, ischemic stroke, congestive heart failure, atrial fibrillation/flutter, or cardiovascular death). The survey detailed the use of NSAIDs, both overall and according to type (ibuprofen, naproxen, or diclofenac). NSAIDs were not associated with an increased cardiovascular-event rate when used in gout patients. Ibuprofen and naproxen seemed to have better cardiovascular risk profiles than diclofenac [43]. Similarly, according to our study, heart failure was reported for ibuprofen with a lower probability than that reported for diclofenac.

Our results show that the probability of tachycardia being reported as an adverse reaction for ibuprofen is higher than the probabilities reported for other NSAIDs (aspirin, diclofenac, ketoprofen, celecoxib, and etoricoxib). Cardiovascular ADRs can be linked to the mechanism of action of NSAIDs (the inhibition of COX enzymes) and the entire cascade of effects that negatively impact the cardiovascular system: pro-thrombotic effects; blood pressure elevation; fluid retention; worsening heart failure, especially in elderly people, etc. [44,45,46]. In a study utilizing thirty-eight hearts removed from New Zealand white rabbits and perfused using a Langendorff setup, electrophysiology studies were performed to investigate the action potential duration at 90% of repolarization (APD90), QT intervals, and effective refractory period (ERP). Perfusion with ibuprofen led to an increased incidence of ventricular arrhythmias, higher than that observed in the case of diclofenac. Also, a higher dose of ibuprofen increases the risk of arrhythmia [47]. Moreover, there are other studies, e.g., Yang et al. (2008), in which the authors observed raised arrhythmia susceptibility in a guinea pig model with ibuprofen [48]. These findings raise the awareness of the increased arrhythmia susceptibility, based on altered cardiac electrophysiology, in patients treated with NSAIDs. Patients with known QT shortening should be monitored, even strictly, during NSAID treatment. Some case studies, e.g., the case of a 13-year-old girl who experienced palpitations following regular doses of ibuprofen (after the third 400 mg dose of ibuprofen) suggest possible arrhythmogenic mechanisms associated with ibuprofen [49]. This could be a consequence of ibuprofen’s reductions of the action’s potential duration and the effective refractory period, along with the associated decrease in the propagation of electrical excitation in the heart, which sets up a substrate for arrhythmia [48].

Concerning the evaluation of the atrial fibrillation or flutter associated with NSAIDs, Schmidt et al., in a case–control study using data from medical databases (Northern Denmark), stated that no NSAIDs were associated with a lower risk than ibuprofen, and diclofenac, in particular, conferred a higher risk. The increased effect estimates associated with the use of the individual NSAIDs remained elevated for both high-dose and low-dose tablets. High-dose tablets of ibuprofen, naproxen, and diclofenac, however, were associated with higher risks than low-dose tablets [50].

By comparison, the probability of reported myocardial infarction (MI) as an ADR associated with ibuprofen is higher than the values for ketoprofen and nimesulide. A case–control study was performed studying a cohort of new NSAID users (1999–2011). The data for this study was obtained from six different longitudinal population-based healthcare databases from four European countries and covered a population of 32 million subjects. The myocardial infarction risk was lower for ibuprofen in comparison to ketorolac and, similar to our study, higher than the risks for naproxen, nimesulide, and ketoprofen [51]. Andersohn et al. conducted a case–control study utilizing a cohort of 486,378 participants with an age of at least 40 years who were registered in the UK General Practice Research Database and who had at least one prescription for an NSAID between 1 June 2000 and 31 October 2004. Myocardial infarction was matched in 3643 cases from this group with 13,918 controls, based on age, sex, general practice, and year of cohort entry. At ibuprofen doses of 1200 mg/day, the risk of MI was slightly lower than for naproxen. Still, the reported risk of MI was not significant for either ibuprofen or naproxen across the dose ranges studied [52].

Also, cardiac arrest seems to be reported as being more probable with ibuprofen, in comparison to ketoprofen, nimesulide, and etoricoxib. In a study that included data from the nationwide Danish Cardiac Arrest Registry, Sondergaard et al. showed that ibuprofen was associated with a significantly increased risk of out-of-hospital cardiac arrest. By comparison, naproxen, celecoxib, and rofecoxib proved to be not as significantly associated with increased risk of out-of-hospital cardiac arrest [53]. Our study could not include information addressing whether the cardiac event (cardiac arrest) occurred out of hospital or not.

Regarding stroke, several studies have been reported, including one conducted as part of the EU-funded project “Safety of Non-Steroidal Anti-Inflammatory Drugs” (SOS). The study evaluated the risks associated with the use of an individual NSAID, using data from six healthcare databases across four European countries. Among the NSAIDs evaluated, the highest significant risk of ischemic stroke was observed for ketorolac, but significantly increased risks for diclofenac, indomethacin, rofecoxib, ibuprofen, nimesulide, diclofenac with misoprostol, and piroxicam were also reported. The ischemic stroke risk associated with NSAID use was generally observed to be higher in persons of younger age, males, and those with a prior history of stroke [54]. According to the present study, by contrast, stroke is potentially associated with ibuprofen at a lower probability relative to acetylsalicylic acid, meloxicam, celecoxib, and etoricoxib. This finding may be linked to prescription patterns associated with coxibs and meloxicam when used for chronic conditions (e.g., arthritis), a usage potentially increasing cumulative thrombotic risk due to long-term use and high doses [55,56,57].

Concerning the risk of heart failure associated with NSAIDs, Arfè A. et al. (2016) found, in five population-based healthcare databases from four European countries (the Netherlands, Italy, Germany, and the United Kingdom), an increased risk of hospital admission for heart failure in association with current individual usage of several traditional NSAIDs (diclofenac, ibuprofen, indomethacin, ketorolac, naproxen, nimesulide, piroxicam, and possibly nabumetone) and two COX-2 inhibitors (etoricoxib and rofecoxib) [58]. Taking into account other associated pathologies, a study correlated the risk of new-onset heart failure hospitalization in patients with type 2 diabetes mellitus (using a Danish national database) and concluded that NSAIDs, particularly diclofenac and ibuprofen, were both widely used and associated with an increased risk of new-onset heart failure hospitalization in patients with type 2 diabetes mellitus [59]. By contrast, our study did not reveal a higher probability of reported heart failure associated with ibuprofen, compared to other NSAIDs, and with the particular case of associated type 2 diabetes, no correlation could be made. Moreover, a lower probability of reported heart failure was noticed in comparisons with acetylsalicylic acid, diclofenac, meloxicam, piroxicam, etoricoxib, and celecoxib.

Thrombosis was reported for ibuprofen with a higher probability, in comparison to ketoprofen. Another research project, performed by Stichtenoth et al., showed that ketoprofen was more effective in the inhibition of platelet aggregation and platelet thromboxane synthesis than ibuprofen [60]. Also, our study revealed a lower probability of reported thrombosis for ibuprofen, when compared to acetylsalicylic acid. This may be a consequence of the dose-dependent indications of acetylsalicylic acid [61] or the resistance to the antithrombotic action of low doses of this drug, as reported by Gum et al. [62]. Considering the clinical safety implications associated with the administration of NSAIDs, the selection of a proper drug must account for several factors, like COX-2 selectivity, pharmacokinetics (i.e., half-life, absorption, and metabolism), bioavailability, clinical efficacy, potency and risk potential [29,63,64].

While regulatory authorities have highlighted the cardiovascular risks associated with high-dose ibuprofen (>2400 mg/day) [12], our study could not assess dose-specific risks, due to limitations in the available data. Nonetheless, the overall lower probability of reported cardiovascular ADRs associated with ibuprofen, compared to the other NSAIDs in our sample, may reflect real-world usage patterns, exposure levels, or differences in the underlying patient populations.

### 3.3. Limitations of the Study

This study comprises the preferred terms (PTs) related to cardiovascular adverse reactions concerning ibuprofen reported in EV. Since not all the existing terms and ADR reports related to ibuprofen are available through this database, the precision of the study might be limited. The underreporting of ADRs is a widespread phenomenon, with the number of reported ADRs being below the number of ADRs that occur. The statistics available through the EV database, and other pharmacovigilance systems, only reflect the data on ADRs that were reported, and these statistics therefore cannot be considered exhaustive. The reported ADRs or case reports depend on several factors, such as the awareness of the patient, the source of information (healthcare professionals or other individuals), the nature of the reaction, the administration of other drugs, the conditions in which the drug was used, etc.

The safety profiles observed could be limited in their precision; thus, the data cannot be used to determine the frequency of ADRs because the spontaneous cases reported in EV are based on a suspicion of causality, which does not necessarily mean that a causal relationship has been established. One major limitation of this study is the lack of information on drug dosage and treatment duration for the majority of spontaneous reports. This constrains our ability to analyze dose–response relationships, which are particularly important for ibuprofen, since its cardiovascular risk is known to be dose-dependent. As with all pharmacovigilance-based analyses, the findings should be interpreted in the context of reporting bias, missing data, and the inability to establish causality.

The sales and actual use levels of each studied drug are not provided by EV; thus, the exact number of patients who received the drugs cannot be determined by using this tool. The calculated ROR cannot be used for the quantitative determination of the ADR risk for ibuprofen because it only indicates the potential safety issues that might occur.

## 4. Materials and Methods

### 4.1. Study Design

A pharmacovigilance-based study of ADRs reported in the EV database for ibuprofen from 27 January 2003 (the date of the first report) until 11 May 2025 was performed [14]. A descriptive analysis of all cases reported in EV was conducted, and subsequently, a disproportionality analysis of cases with respect to certain PTs referring to cardiovascular toxicity was conducted [15]. The disproportionate results were evaluated by comparison to ADRs reported for other NSAIDs (diclofenac, ketoprofen, naproxen, ketorolac, meloxicam, piroxicam, nimesulide, acetylsalicylic acid, ibuprofen + pseudoephedrine, celecoxib, and etoricoxib). All data were extracted on 17 May 2025.

### 4.2. Material

ADRs are collected in EV based on ICSRs, which include general characteristics (age, sex, national origin, and reporter category). Several age categories were used in these ICSRs (0–1 month, 2 months–2 years, 3–11 years, 12–17 years, 18–64 years, 65–85 years, more than 85 years, or NS). EMA regulations mention three categories for reporting sex (male, female, or NS), three for national origin (EEA, non-EEA, or NS), and three for reporter category (healthcare professional—HP, non-HP, or NS [16]. For reported ADRs, the MedDRA includes five levels of hierarchy: (i) SOC, (ii) High-Level Group Terms (HLGT), (iii) High-Level Terms (HLT), (iv) PT, and (v) Lowest-Level Terms (LLT); the last is the most granular. In EV, the number of cases is provided at the PT level, and a PT may be associated with one or more SOCs. Moreover, to facilitate the investigation of drug safety, MedDRA includes SMQs, which are groups of terms related to a medical condition [17]. A serious ADR is an adverse reaction that results in death, is life-threatening, requires hospitalization or prolongation of existing hospitalization, results in persistent or significant disability or incapacity, or is a birth defect [18].

### 4.3. Pharmacovigilance Analysis

The general characteristics of the ICSRs submitted in EV for ibuprofen were analyzed [19]. Subsequently, the distribution of cases according to the 27 SOCs was assessed. Related to cardiovascular toxicity, 10 SMQs were selected. These groups comprised 40 PTs associated with three SOCs (Table 1) [20].

The disproportionality analysis identifies the probability of the reported ADRs for a drug in comparison to the entire database or to those of other drugs from the same therapeutic areas. For cases reported in EV, the EMA recommends calculating the ROR and 95% CI in order to establish the disproportionate nature of the results. The minimum number of cases is five, and the lower limit of 95% CI has to be higher than 1 [21,22]. To calculate ROR and 95% CI, the MedCalc application (retrieved from https://www.medcalc.org/calc/odds_ratio.php, accessed on 31 May 2025) (Version 23.2.6) was used [23].

$$ROR=\frac{a/b}{c/d}$$

a—The number of individual cases that list the medicinal product of interest and the adverse event of interest;
b—The number of individual cases that list the medicinal product of interest, but not the adverse event of interest;
c—The number of individual cases that list the adverse event of interest but not the medicinal product of interest;
d—The number of individual cases that do not list the adverse event of interest or the medicinal product of interest [24].

### 4.4. Ethics

This study does not utilize any personally identifiable patient information. Moreover, the research does not fall under the scope of human-subject research regulations or the data protection guidelines. Therefore, no ethical approval was required.

## 5. Conclusions

Our study had the goal of assessing the real-world impact of ibuprofen regarding cardiovascular safety, as reflected in a pharmacovigilance database (EV). Even though the numbers of reported ADRs pertaining to the cutaneous and gastrointestinal areas were higher, the seriousness of the reported ADRs within the cardiovascular safety area was higher, and therefore we considered the necessity for further analysis to be more stringent. Although the probability values for reported myocardial infraction and cardiac arrest were higher for ibuprofen than for ketoprofen and nimesulide, for some SMQs like heart failure, embolism, and stroke, our study doesn’t show a higher probability of reports associated with ibuprofen relative to the comparator drugs. Overall, according to our study, the probability of the reported cardiovascular adverse reactions associated with ibuprofen is lower than for the rest of the NSAIDs. Other clinical investigations have to be performed to confirm these results and to better understand the cardiovascular safety profile of ibuprofen.

## Figures and Tables

**Table 1:** SMQs selected for assessing the cardiovascular toxicity. PT—Preferred Terms; SMQ—Standardized MedDRA Queries; SOC—System Organ Class.

SMQ	SOC	PT
Stroke	Nervous system disorders	Embolic stroke
	Vascular disorders	Embolic stroke
	Nervous system disorders	Hemorrhagic stroke
	Vascular disorders	Hemorrhagic stroke
	Nervous system disorders	Ischemic stroke
	Vascular disorders	Ischemic stroke
	Nervous system disorders	Lacunar stroke
	Vascular disorders	Lacunar stroke
	Nervous system disorders	Thrombotic stroke
	Vascular disorders	Thrombotic stroke
Thrombosis	Vascular disorders	Thrombosis
	Vascular disorders	Arterial thrombosis
	Vascular disorders	Deep vein thrombosis
	Vascular disorders	Venous thrombosis
Embolism	Vascular disorders	Embolism
	Vascular disorders	Embolism arterial
	Vascular disorders	Embolism venous
Hypertension	Vascular disorders	Hypertension
	Vascular disorders	Accelerated hypertension
	Vascular disorders	Diastolic hypertension
	Vascular disorders	Malignant hypertension
	Vascular disorders	Nocturnal hypertension
	Vascular disorders	Orthostatic hypertension
	Nervous system disorders	Orthostatic hypertension
	Vascular disorders	Systolic hypertension
Hypertensive emergency	Vascular disorders	Hypertensive emergency
Hypertensive urgency	Vascular disorders	Hypertensive urgency
Tachycardia	Cardiac disorders	Tachycardia
	Cardiac disorders	Atrial tachycardia
	Cardiac disorders	Sinus tachycardia
	Cardiac disorders	Supraventricular tachycardia
	Cardiac disorders	Ventricular tachycardia
Myocardial infarction	Cardiac disorders	Myocardial infarction
	Vascular disorders	Myocardial infarction
	Cardiac disorders	Acute myocardial infarction
	Vascular disorders	Acute myocardial infarction
Cardiac arrest	Cardiac disorders	Cardiac arrest
Cardiac failure	Cardiac disorders	Cardiac failure
	Cardiac disorders	Cardiac failure acute
	Cardiac disorders	Cardiac failure congestive

## Data Availability

The data are contained in the article and Supplementary Materials.

## Supplementary Materials

The following supporting information can be downloaded at: https://www.mdpi.com/article/10.3390/ph18071045/s1, Table S1: Number of ADRs reported for selected SMQs.
