# Supplementary material for: Exploring the Cardiovascular Safety Profile of Ibuprofen: Insights from EudraVigilance Database
Source: Pharmaceuticals (Basel). 2025 Jul 17;18(7):1045. doi: 10.3390/ph18071045 (PMC12300713; doi:10.3390/ph18071045)
Supplement: Supplementary file 1 [file pharmaceuticals-18-01045-s001.zip › pharmaceuticals-3733322-supplementary.pdf]

Supplementary Material

Table S1. Number of ADRs reported for selected SMQs. ADRs – adverse drug reactions; SMQs - Standardized MedDRA Queries.

	**Stroke**	**Thrombosis**	**Embolism**	**Hypertension**	**Hypertensive emergency**	**Hypertensive urgency**	**Tachycardia**	**Myocardial infarction**	**Cardiac arrest**	**Heart failure**
Ibuprofen	49	157	9	613	14	3	736	255	227	168
Ibuprofen + Pseudoephedrine	6	6	6	16	0	0	14	8	3	1
Acetylsalicylic acid	1634	893	74	873	6	4	841	1797	503	793
Diclofenac	35	153	20	1183	1	1	518	341	173	287
Ketoprofen	16	14	2	148	0	0	15	29	34	51
Naproxen	28	73	6	692	1	0	472	312	112	96
Ketorolac	3	19	3	125	1	0	63	21	44	14
Meloxicam	18	21	1	108	0	0	53	91	23	43
Piroxicam	1	5	0	67	0	0	27	11	4	15
Nimesulide	5	2	1	39	0	0	56	9	6	8
Celecoxib	276	295	24	888	1	0	214	908	79	327
Etoricoxib	26	30	5	327	0	2	105	251	20	193

Orange cells – the number of ADRs have been < 5 (minimum number of ADRs established to calculate the disproportionate signal)
